# Drug-Induced Reaction With Eosinophilia and Systemic Symptoms: A Review

**DOI:** 10.7759/cureus.35701

**Published:** 2023-03-02

**Authors:** Moteb Alotaibi

**Affiliations:** 1 Medicine, Unaizah College of Medicine and Medical Sciences, Qassim University, Unaizah, SAU

**Keywords:** ideosyncrasy, cutaneous reaction, eosinophilia, hypersensitivity, drug reaction

## Abstract

Drug-induced reaction with eosinophilia and systemic symptoms (DRESS) is a part of severe cutaneous adverse reactions (SCAR), often a life-threatening condition. DRESS is an uncommon reaction; however, it is more prevalent than Stevens-Johnson syndrome/toxic epidermal necrolysis (SJS/TEN) and is left undiagnosed due to its atypical clinical presentation. So far, no standard criteria or investigational tool assists in early and accurate diagnosis. The administration of systemic corticosteroids remains the widely used first line of management. However, new studies have revealed other potential treatment armamentariums. Because of the potential life-threatening outcome, every physician who deals with acute cases should be familiar with the clinical presentation and be able to start the necessary measurements. Recent studies revealed important information in the pathogenesis and management of the disorder were summarized in this review.

## Introduction and background

Drug-induced reaction with eosinophilia and systemic symptoms (DRESS) is a severe, life-threatening systemic drug reaction that potentially involves multiple organs, including the skin. The syndrome was first described by Bocquet in 1996; since then, many terms have been used to describe this clinical entity, such as drug-induced hypersensitivity syndrome (DIHS), mononucleosis-like syndrome [1,2].

DIHS is now considered an umbrella term that includes all drug-induced reactions with or without cutaneous involvement [3]. The prevalence of DRESS varies considering its diverse clinical manifestations, but it is estimated to be 2.18 per 100,000 patients, with a slight female predominance (55%) in the United States [4]. The major drug groups that cause these reactions include antibiotics (74%) and antiepileptics (20%) [4,5]. DRESS syndrome is relatively more common than Stevens-Johnson syndrome/toxic epidermal necrolysis (SJS/TEN), and approximately 95% of DRESS patients require hospitalization, where the mortality is found to be 3%. The healthcare cost of DRESS for in-hospital management is at least USD$17,000 per patient [4]. These underscore the economic burden of the disease on the health system.

## Review

Methodology

A literature search was conducted in the PubMed database using the following key terms: ‘Drug-induced reaction with eosinophilia and systemic symptoms’ or ‘DRESS’ or ‘Drug hypersensitivity syndrome’. We reviewed the titles and abstracts; the most recent and comprehensive papers published only in English were selected. We summarized the topic components into a single article to save the time of busy physicians.

Pathogenesis

DRESS is a T-cell-mediated delayed-type hypersensitivity reaction to a drug or drug metabolites. The pathogenesis is not yet completely understood and is estimated to be a multifactorial process of interaction between drugs and the body’s immune system and metabolism [1]. Recent advances in pharmacogenomics have revealed an association between human leucocyte antigen (HLA) haplotypes and susceptibility to react with certain drugs [1,3]. It has been hypothesized that any alteration in body metabolism that affects the pharmacokinetics, as in the case of kidney or liver diseases, will alter drug metabolite levels and predispose patients to DRESS [1,3]. Certain cytokines have been found to play a major role in the pathogenesis, such as interleukin (IL)-5, which is responsible for peripheral eosinophilia, thymus- and activation-regulated chemokine (TARC), and macrophage-derived chemokine (MDC) [6]. In some patients exhibiting DRESS, Herpesviruses, such as human herpes virus 6 (HHV6) and 7 (HHV7), Epstein-Barr virus (EBV), and cytomegalovirus (CMV), have been detected in their samples. Approximately 76-80% of cases were found to test positive for HHV6, HHV7, or EBV, suggesting that viral infection-induced reactivation may contribute to disease development [7]. Reactivation is usually detected two-four weeks after onset, which may be associated with symptom relapse after initial improvement [8]. The exact pathogenic role remains unclear [3,6,9]. Recently, activation of the JAK-STAT (Janus kinases-signal transducer and activator of transcription proteins) signaling pathway in a refractory DRESS syndrome case was reported [10]. Moreover, IL-5 possibly plays a critical role in DRESS pathophysiology [11]. The causative drug can be traced in 88% of cases [12,13].

Clinical features

DRESS presents with a wide range of clinical features, which often leads to delays in accurate diagnosis. Typical clinical features include a sudden onset of skin rash along with fever within three weeks to three months duration after the initiation of the culprit drug [9]. Fever of ≥38.5°C is present in ≥90% of patients; lymphadenopathy occurs in 50-75% of patients; hematologic abnormality occurs in ≥90% in the form of eosinophilia (average 3.5 × 10^9^/L), and atypical lymphocytosis occurs in around 65-80% of patients [3]. Skin rash is the most common clinical sign, presenting in 99-100% of patients, particularly the polymorphous maculopapular rash (in 85% of patients), sparing the palms, soles, and mucous membranes [9,12]. However, DRESS may also present in other forms, such as erythematous macules, pustules, target-like, eczema-like lesions, or purpuric rash [9]. Facial edema is present in 76% of cases and has been correlated with the severity of the disease [8]. The rash commonly spares the palms and soles but mucous membranes may be affected in 56% of cases, although milder than in SJS/TEN syndrome [3,7]. The most alarming sign of DRESS is the involvement of internal organs. In a large prospective study, 91% of patients had visceral organ involvement. The liver was majorly affected, i.e., in 75% of cases and cholestatic, mixed, and hepatocellular types of liver injury have been reported [7]. Kidneys were involved in 37% and lungs were involved in 32% of patients [13]. The Registry of Severe Cutaneous Adverse Reaction [RegiSCA] scoring system is a widely accepted method for diagnosing DRESS and has been reported to be more sensitive than the Japanese consensus group criteria [13,14].

Investigations

The diagnosis of DRESS syndrome should be considered in any patient with a recent history of the intake of new medication i.e., two-eight weeks, and presents with a combination of skin eruption and fever. In suspected cases, it is highly recommended to conduct a series of comprehensive laboratory investigations and monitor frequently. These preliminary investigations should include, at least, a complete blood count with differential and peripheral blood smear evaluating for eosinophilia, the presence of atypical lymphocytes and other hematologic abnormalities, and liver and kidney function tests. Frequent testing, even if the initial results are normal, is of crucial importance since the detection of laboratory signs of visceral involvement may be delayed behind cutaneous manifestations; even eosinophilia may be delayed for 1-2 weeks [3,9]. Additional tests are recommended according to clinical indications. To date, there is no strong recommendation for viral testing. A liver injury should be assessed and severity must be staged according to the DILI Expert Working Group [15]. Choudhary et al. found that a combination model of total body surface area at baseline, eosinophil count, and high-sensitivity C-reactive protein demonstrated a sensitivity of 96% and a specificity of 100% in the prediction of DRESS [16].

Serum thymus and activation-regulated chemokine (TARC) levels exhibit a strong correlation with blood eosinophil count and were found to have 100% sensitivity and 92.3% specificity in the diagnosis of DRESS syndrome, with a threshold value of 13,900 pg/mL [17]. Mizukawa et al. established a scoring system that can identify high-risk patients by stratifying them into three risk categories: low, intermediate, and high [18]. Research on biomarkers for severe drug reactions has been rapidly evolving, and seven miRNAs have been identified to date as potential biomarkers for identifying DRESS [19].

Management

The first step in the management of the disease is to identify the culprit drug and limit its usage as well as any other cross-reacting drug. Understandably this is challenging, particularly with polypharmacy patients. So far, there is no gold standard method to identify the culprit drug during the active reaction and is solely dependent on a physician's search for known high-risk medications with relevant chronological relationships. Therefore, a multidisciplinary team depending on the involved organs is critical to facilitating accurate diagnosis, monitoring, and treatment. As there are no standard treatment guidelines for DRESS management, many existing treatments have been used. Systemic steroids are the widely used first line of management [3,8,20]. The alternate therapeutic options that have demonstrated favorable outcomes, as mentioned in case reports and small-scale studies, include steroid-sparing medications such as cyclosporine, intravenous immunoglobulin (IVIG), mycophenolate mofetil, cyclophosphamide, and rituximab [7]. The Spanish guideline recommended cyclosporine as a second-line therapy in the absence of control or contraindication of corticosteroids [20]. Several studies have demonstrated the efficacy of cyclosporin in the management of DRESS syndrome. In a case report by Hashizume et al., it has been stated that six cases of DRESS syndrome were treated successfully with cyclosporin without relapse [21]. In a prospective case-control study, cyclosporin was associated with a shorter time of cessation of rash progression, rash resolution, and normalization of leukocytosis, eosinophilia, and hepatocellular markers in comparison with a corticosteroid-treated group [22]. Cyclosporine has a beneficial effect on DRESS syndrome, given its rapid onset of action, and effective inhibition of T cell proliferation and IL-5 production, which is responsible for the development of eosinophilia [3,23]. Kim et al. reported a steroid-refractory DRESS case that was treated successfully with the drug tofacitinib. They proposed that JAK inhibitors could be potential therapeutic targets in treating DiHS/DRESS [10]. IL-5 and IL-5 receptor-targeting monoclonal antibodies show great promise in DRESS management [11].

## Conclusions

Early recognition of signs of DRESS and cessation of the suspected medication(s) is critical to prevent the associated mortality and morbidity. Cyclosporin offers promising outcomes in the management of DRESS with good tolerability. These results must be assessed further in well-designed clinical trials.
